# Systems biology of myasthenia gravis, integration of aberrant lncRNA and mRNA expression changes

**DOI:** 10.1186/s12920-015-0087-z

**Published:** 2015-03-18

**Authors:** ZhaoHui Luo, Ye Li, XiaoFang Liu, MengChuan Luo, LiQun Xu, YueBei Luo, Bo Xiao, Huan Yang

**Affiliations:** Department of Neurology, Xiangya Hospital, Central South University, Changsha, 410008 China; Department of Neurology, Fujian Medical University Union Hospital, Fuzhou, 350001 China

**Keywords:** Long non-coding RNAs (lncRNAs), Myasthenia gravis(MG), Thymoma, Cis, Transcription factors

## Abstract

**Background:**

A novel class of transcripts, long non-coding RNAs (lncRNAs), has recently emerged as a key player in several biological processes, and important roles for these molecules have been reported in a number of complex human diseases, such as autoimmune diseases, neurological disorders, and various cancers. However, the aberrant lncRNAs implicated in myasthenia gravis (MG) remain unknown. The aim of the present study was to explore the abnormal expression of lncRNAs in peripheral blood mononuclear cells (PBMCs) and examine mRNA regulatory relationship networks among MG patients with or without thymoma.

**Methods:**

Microarray assays were performed, and the outstanding differences between lncRNAs or mRNA expression were verified through RT-PCR. The lncRNAs functions were annotated for the target genes using Gene Ontology (GO) and the Kyoto Encyclopedia of Genes and Genomes (KEGG) biological pathway. The potential regulatory relationships between the lncRNAs and target genes were analyzed using the ‘cis’ and ‘trans’ model. Outstanding lncRNAs were organized to generate a TF-lncRNA-gene network using Cytoscape software.

**Results:**

The lncRNA and mRNA expression profile analysis revealed subsets of differentially expressed genes in MG patients with or without thymoma. A total of 12 outstanding dysregulated expression lncRNAs, such as lncRNA oebiotech_11933, were verified through real-time PCR. Several GO terms including the cellular response to interferon-γ, platelet degranulation, chemokine receptor binding and cytokine interactions were very important in MG pathogenesis. The chromosome locations of some lncRNAs and associated co-expression genes were demonstrated using ‘cis’ analysis. The results of the ‘trans’ analysis revealed that some TFs (i.e., CTCF, TAF1and MYC) regulate lncRNA and gene expression. The outstanding lncRNAs in each group were implicated in the regulation of the TF-lncRNA-target gene network.

**Conclusion:**

The results of the present study provide a perspective on lncRNA expression in MG. We identify a subset of aberrant lncRNAs and mRNAs as potential biomarkers for the diagnosis of MG. The GO and KEGG pathway analysis provides an annotation to determine the functions of these lncRNAs. The results of the ‘cis’ and ‘trans’ analyses provide information concerning the modular regulation of lncRNAs.

**Electronic supplementary material:**

The online version of this article (doi:10.1186/s12920-015-0087-z) contains supplementary material, which is available to authorized users.

## Background

Myasthenia gravis (MG) is a T and B cell-mediated autoimmune disease of the neuromuscular junction; cytokines and chemokines may play a crucial role in the pathogenesis and perpetuation of this disease [1-3]. Muscle weakness and fatigue, as hallmarks of MG, indicate improper signaling between T and B cells, resulting in the elicitation of an antibody-mediated autoimmune response against the acetylcholine receptor (AChR) located at neuromuscular junctions [4,5]. In most cases, autoantibodies against the AChR are detected. Recently, other targets, such as muscle-specific kinase (MuSK) and lipoprotein-related protein 4 (LRP4), have been described [3]. However, the mechanisms that contribute to the development or pathogenesis of MG are highly complex. A puzzling yet interesting characteristic of MG is that many patients show thymic abnormalities, such as thymic germinal center hyperplasia, whereas other patients develop thymic tumors [6-9]. The thymus has long been considered to be closely associated with the pathogenesis of MG, and some studies have suggested that MG autoimmune reactions originate in the thymus [7,10]. The relationship between MG and thymoma has been repeatedly suggested; however, many questions and controversies still remain. The biological and clinical behavior of thymomatous MG following thymectomy has not been adequately investigated, and evidence is lacking concerning both the clinical course of MG and the prognosis of thymomas associated with MG following surgical management [7,11]. Therefore, additional studies concerning the role of the thymus gland in the pathogenesis of MG are required.

Long non-coding RNAs (lncRNAs) are a group of RNA transcripts that are more than 200 nucleotides in length and lack significant open reading frames (ORFs) [12-15]. lncRNAs have only been recently identified, and most of the functions of these molecules remain unclear. The long nucleotide chain of lncRNAs can either form a complex spatial structure that interacts with protein factors or provide a large region for the concurrent binding of many molecules that collectively participate in genomic imprinting, X-chromosome silencing, chromosome modification, intranuclear transport, and transcriptional activation and interference [13,14,16-18],thereby regulating cell growth, differentiation, development, senescence and death [19-21]. Although several lncRNAs molecules have been implicated in diverse processes and diseases [22,23], only a few examples of the regulation of the immune system through lncRNAs have been described [24-26].

In the present study, we performed an array of lncRNA chip assays on PBMCs of MG patients. The outstanding lncRNAs functions were annotated based on co-expression genes and the GO biological process. The relationships among the lncRNAs were revealed through ‘cis’ and ‘trans’ analyses. These results provide information for further studies on MG.

## Results

### lncRNA and mRNA expression profile in MG

**Figure 1:**
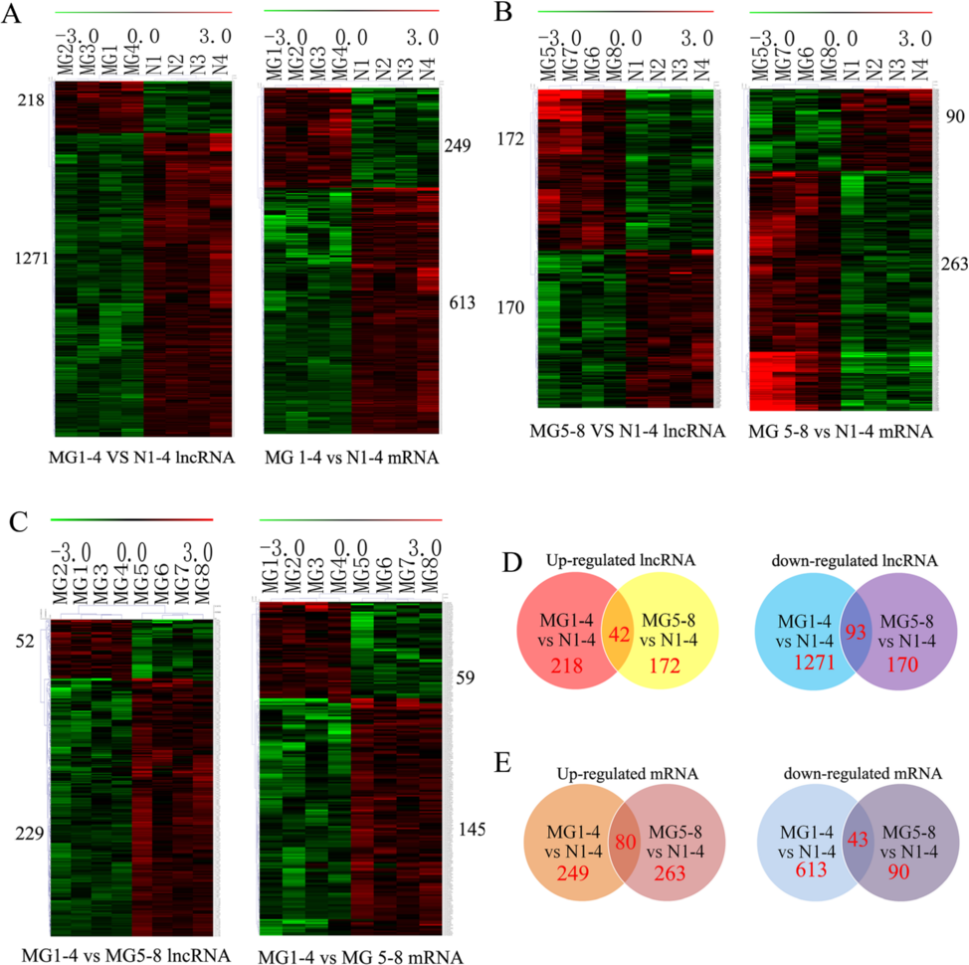
**The differences in cluster heat maps of lncRNA for the three groups. A**: The lncRNA and mRNA cluster heatmaps of MG patients with thymoma compared with healthy controls. **B**: The lncRNA and mRNA cluster heat map of MG patients without thymoma compared with healthy controls. **C**: The lncRNA and mRNA cluster heat map of MG patients with thymoma compared with MG patients without thymoma. **D**: Common differentially expressed lncRNAs between MG patients with or without thymoma. The number of common upregulated lncRNAs is shown on the left, and the number of common downregulated lncRNAs is shown on the right. **E**: Common differentially expressed mRNAs between MG patients with or without thymoma. The number of common upregulated mRNAs is shown on the left, and the number of common downregulated mRNAs is shown on the right. MG1-4 represents the MG with thymoma group, MG5-8 represents the MG without thymoma group, and N1-4 represents the healthy control group.

To investigate the expression levels of lncRNAs associated with MG with or without thymoma, lncRNA and mRNA microarray analyses were performed on the PBMCs of MG patients. After separating the signal from noise and performing a t-test, significant differences in lncRNAs and mRNA expression of up to two-fold (P < 0.05 and FDR < 0.05) were observed. These results are summarized in Table 1, and detailed information is provided in Additional file 1. The lncRNA and mRNA expression data were clustered using Cluster3.0, as shown in Figure 1. Using dendrogram-based methods for clustering, the samples were further separated into two subgroups through hierarchical clustering based on similar expression patterns, and the results indicate that the expression of these lncRNAs and mRNAs was significantly different in MG patients with or without thymoma. This difference could potentially distinguish the disease group from MG patients without thymoma or healthy individuals. We also calculated the common upregulation or downregulation of lncRNA or mRNA expression in MG patients with or without thymoma versus healthy controls. A total of 42 lncRNAs exhibited upregulated expression, and 93 lncRNAs exhibited downregulated expression (Figure 1D), whereas 80 mRNAs exhibited upregulated expression and 43 mRNAs exhibited downregulated expression in the two groups (Figure 1E). The detailed data are provided in Additional file 2. These results suggest that these lncRNAs and mRNAs may have common functions in the development of MG.

**Table 1:** **Dysregulated lncRNAs and mRNAs**

		**mRNA**			**Long non-coding RNA**		
**Experimental group**	**Control group**	**Up-regulated**	**Down-regulated**	**In total**	**Up-regulated**	**Down-regulated**	**In total**
MG1-4^*^	N1-4	249	613	862	218	1271	1489
MG5-8	N1-4	263	90	353	172	170	342
MG1-4	MG5-8	59	145	204	52	229	281

MG1-4 represents the MG with thymoma group, MG5-8 represents the MG without thymoma group, and N1-4 represents the healthy control group.

### The lncRNA function annotation and statistical characteristics network

To further explore the function of lncRNAs in MG, the results of the lncRNA and mRNA chip analyses were subjected to Pearson’s correlation coefficient analysis, in which co-expression was considered at P > 0.8. To determine the level of lncRNA and mRNA co-expression, we divided the different lncRNAs into two subsets: the upregulated lncRNAs and downregulated lncRNAs. When the subset contained more than 100 different lncRNAs, we selected the top 100 most distinguished lncRNAs, and when the subset contained less than 100 lncRNAs, all lncRNAs in the subset were used. Because the file is extremely large, the information pertaining to lncRNA oebiotech_11933 is shown as a representative result. The results for the other lncRNAs are provided in Additional file 3.

lncRNA oebiotech_11933 exhibited the highest upregulation of lncRNA expression among MG patients with thymoma versus healthy controls. A total of 552 genes(e.g., G0S2) relative to lncRNA oebiotech_11933 exhibited P > 90% (Table 2). These genes were further analyzed using DAVID functional annotation software (http://david.abcc.ncifcrf.gov/ gene2gene.jsp). The lncRNA oebiotech_11933 function was annotated using GO and KEGG pathway analyses. Selecting the reliability prediction terms(according to the P-value and enrichment), a total of 20 enrichment GO terms were obtained. Table 3 indicates that lncRNA oebiotech_11933 was associated with transcriptional regulation, cell surface receptor-linked signal transduction, and immune responses, among other terms.

**Table 2:** **Co-expressed genes of lncRNA oebiotech_11933 (only P > 90% genes are shown)**

**Correlation**	**P-value**	**Gene ID**	**Probe**	**Gene symbol**
0.98291	1.12E-08	50486	A_23_P74609	G0S2
0.945413	3.48E-06	2069	A_23_P41344	EREG
-0.94353	4.11E-06	10605	A_23_P207811	PAIP1
0.942434	4.52E-06	23710	A_24_P4816	GABARAPL1
0.93273	9.68E-06	1647	A_23_P23221	GADD45A
0.926358	1.51E-05	64651	A_23_P121011	CSRNP1
-0.9236	1.8E-05	80851	A_33_P3413305	SH3BP5L
0.92047	2.19E-05	64092	A_23_P29005	SAMSN1
0.913331	3.32E-05	64651	A_33_P3224070	CSRNP1
0.912667	3.45E-05	10123	A_33_P3323722	ARL4C
0.911543	3.67E-05	1844	A_24_P37409	DUSP2
0.909644	4.07E-05	124935	A_24_P296508	SLC43A2
0.901592	6.15E-05	392288	A_24_P58037	LOC392288
0.900089	6.62E-05	5142	A_23_P74278	PDE4B

**Table 3:** **GO analysis of lncRNA oebiotech_11933 co-expression genes**

**Term**	**Count**	**%**	**P-value**
GO:0045449 ~ regulation of transcription	92	2.15	0.00213485
GO:0051252 ~ regulation of RNA metabolic process	70	1.64	0.00102494
GO:0006350 ~ transcription	68	1.59	0.0560407
GO:0006355 ~ regulation of transcription, DNA-dependent	67	1.57	0.00236389
GO:0007166 ~ cell surface receptor linked signal transduction	60	1.40	0.07527543
GO:0007242 ~ intracellular signaling cascade	51	1.19	0.00221611
GO:0006955 ~ immune response	47	1.10	6.45E-09
GO:0042127 ~ regulation of cell proliferation	47	1.10	3.29E-07
GO:0006952 ~ defense response	43	1.00	1.57E-08
GO:0009611 ~ response to wounding	41	0.96	2.14E-09
GO:0010033 ~ response to organic substance	39	0.91	3.88E-05
GO:0016265 ~ death	39	0.91	4.21E-05
GO:0008219 ~ cell death	38	0.89	8.13E-05
GO:0031328 ~ positive regulation of cellular biosynthetic process	37	0.86	6.75E-05
GO:0009891 ~ positive regulation of biosynthetic process	37	0.86	9.00E-05
GO:0006915 ~ apoptosis	36	0.84	1.05E-05
GO:0012501 ~ programmed cell death	36	0.84	1.44E-05
GO:0010604 ~ positive regulation of macromolecule metabolic process	35	0.82	0.01188074
GO:0051173 ~ positive regulation of nitrogen compound metabolic process	34	0.79	2.18E-04
GO:0042981 ~ regulation of apoptosis	33	0.77	0.01376469

Moreover, lncRNA oebiotech_11933 KEGG pathways are listed in Table 4, including ‘cytokine-cytokine receptor interaction’, ‘MAPK signaling pathway’, ‘chemokine signaling pathway’, ‘NOD-like receptor signaling pathway’, and ‘Toll-like receptor signaling pathway’. Previous studies have reported that these pathways are associated with lymphocyte immune cell proliferation and cancer development pathways [27-30]. The results of the GO and KEGG pathway analyses consistently showed that lncRNA oebiotech_11933 is associated with lymphocyte immune cell proliferation and cancer development pathways.

**Table 4:** **KEGG pathway analysis of lncRNA oebiotech_11933 co-expression genes**

**Term**	**Count**	**%**	**P-value**
hsa04060:Cytokine-cytokine receptor interaction	20	0.47	2.58E-04
hsa04010:MAPK signaling pathway	15	0.35	0.02771
hsa04062:Chemokine signaling pathway	14	0.33	0.003659
hsa04621:NOD-like receptor signaling pathway	9	0.21	4.56E-04
hsa04620:Toll-like receptor signaling pathway	7	0.16	0.080742
hsa04920:Adipocytokine signaling pathway	6	0.14	0.048983
hsa05219:Bladder cancer	5	0.12	0.035633

**Figure 2:**
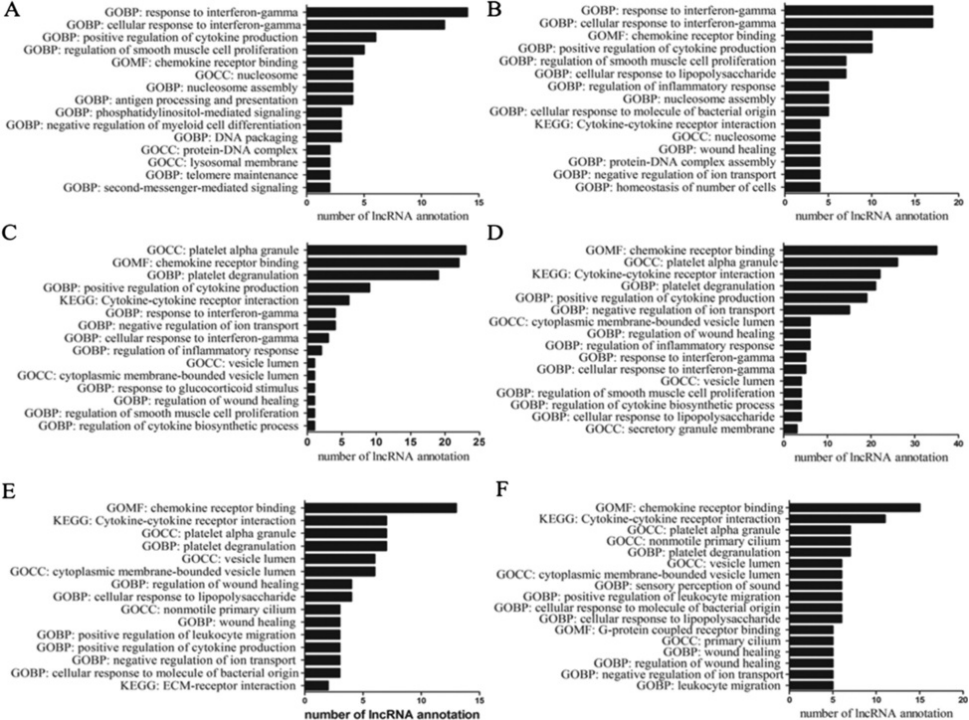
**The top 100 and top 200 GO terms or pathways for the difference lncRNA co-expression genes between the three groups. A-B**: The top 100 and top 200 GO terms or pathways for the difference lncRNAs co-expression genes between MG patients with thymoma and healthy controls. **C-D**: The top 100 and top 200 GO terms or pathways for the difference lncRNA co-expression genes between MG patients without thymoma and healthy controls. **E-F**: The top 100 and top 200 GO terms or pathways for the difference lncRNA co-expression genes between MG patients with thymoma and MG patients without thymoma.

Furthermore, the lncRNA co-expression genes in each group were also analyzed using DAVID functional annotation software. The results of the GO and KEGG pathway analyses for lncRNA oebiotech_11933 are presented. In addition, the aberrant lncRNA genes were subjected to GO and KEGG pathway analyses. We selected the top 100 and 200 reliability prediction terms(according to the P-value and enrichment) for co-expressed and aberrant lncRNA genes, respectively (Figure 2). In MG patients with thymoma versus healthy controls, the top 100 terms in the GO and KEGG pathway analyses were associated with ‘cellular response to interferon-γ’, ‘positive regulation of cytokine production’, ‘smooth muscle cell proliferation’, and ‘cytokine receptors’, among other terms (Figure 2A). The top 200 terms in the GO and KEGG pathway analyses were associated with cellular responses to interferon-γ (Figure 2B). The 7 most common GO terms are shown in Figures 2A and 2B. The terms ‘cellular response to interferon-γ’, ‘chemokine receptor binding’ and ‘positive regulation of cytokine production’ were the most enriched GO terms. In MG patients without thymoma versus healthy controls, GOCC platelet alpha granule and GOMF chemokine receptor binding were the most enriched among the top 200 GO terms. A total of 14 common GO terms are shown in the Figures 2C and 2D. ‘chemokine receptor binding’, ‘cytokine-cytokine receptor interaction’, and ‘platelet alpha granule’ were the most enriched GO terms. In the MG with thymoma group versus the MG without thymoma group, ‘chemokine receptor binding’ and ‘cytokine-cytokine receptor interaction’ were the most enriched among the top 200 GO terms. A total of 13 common GO terms are shown in Figures 2E and 2F. ‘chemokine receptor binding’ and ‘cytokine-cytokine receptor interaction’ were the most enriched GO terms. The results of the GO and KEGG pathway analyses confirmed that lncRNAs play important roles in the lymphocyte immune system, such as inflammation, cell differentiation and proliferation.

### Validation of disturbed lncRNA expression

**Figure 3:**
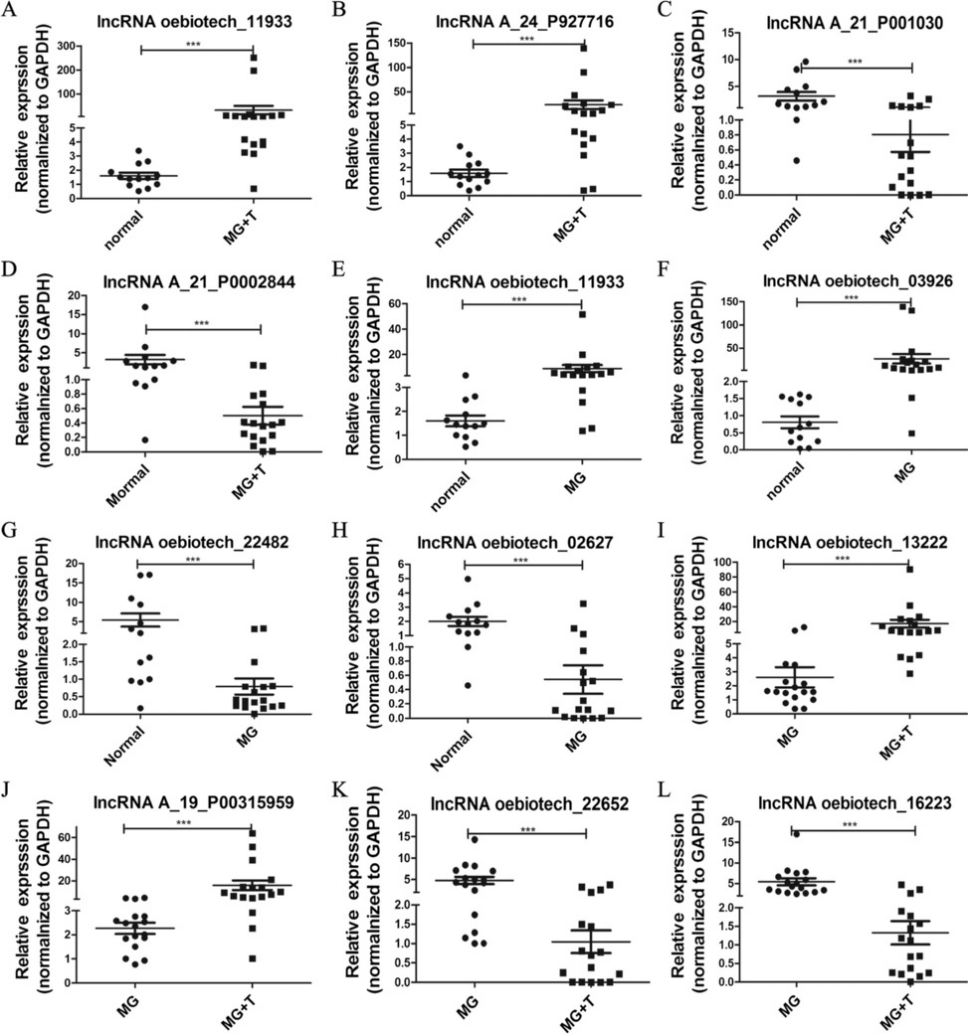
**Validationof the differences in the lncRNAs. A-D**: The 4 different lncRNAs for MG patients with thymoma compared with healthy controls. lncRNA oebiotech_11933 was the most elevated (19.96-fold higher), followed by lncRNA A_24_P927716 (14.75-fold higher), whereas lncRNA A_21_P0010030 and lncRNA A_21_P0002844 were 3.94- and 6.43-fold lower, respectively. All differences were significant. **E-H**: The 4 differences in lncRNAs for MG patients without thymoma compared with healthy controls. lncRNA oebiotech_11933 and lncRNA oebiotech_03926 were 5.45- and 33.1-fold higher, respectively, whereas lncRNA oebiotech_02627 and lncRNA oebiotech_22482 were 3.68-and 6.85-fold lower, respectively; all P-values < 0.05. **I-L**:The 4 differences in lncRNAs for MG patients with thymoma compared with MG patients without thymoma. lncRNA A_19_P00315959 and lncRNA oebiotech_13222 were 6.99- and 6.46-fold higher, respectively, whereas lncRNA oebiotech_22652 and lncRNA oebiotech_16223 were 4.58-and 4.08-fold lower, respectively; all P-values < 0.05.

To verify the disruption of lncRNA expression in MG patients, real-time PCR was performed to examine the up-or downregulation of lncRNAs in each group. As shown in Figures 3A-3D, differences in the expression of 4 lncRNAs were detected in MG patients with thymoma compared with healthy controls: lncRNA oebiotech_11933 was the most elevated (19.96-fold higher expression), followed by lncRNA A_24_P927716 (14.75-fold higher expression), whereas lncRNA A_21_P0010030 and lncRNA A_21_P0002844 exhibited 3.94- and 6.43-fold lower expression, respectively. Figures 3E-3H show the differences in the expression of 4 other lncRNAs in MG patients without thymoma compared with healthy controls. Figures 3I-3L showthe differences in the expression of 4 other lncRNAs in the MG with thymoma group compared with the MG without thymoma group. These results were consistent with the results obtained from the microarray chip analysis.

### ‘Cis’ analysis of the expression of lncRNAs and adjacent co-expression genes

**Figure 4:**
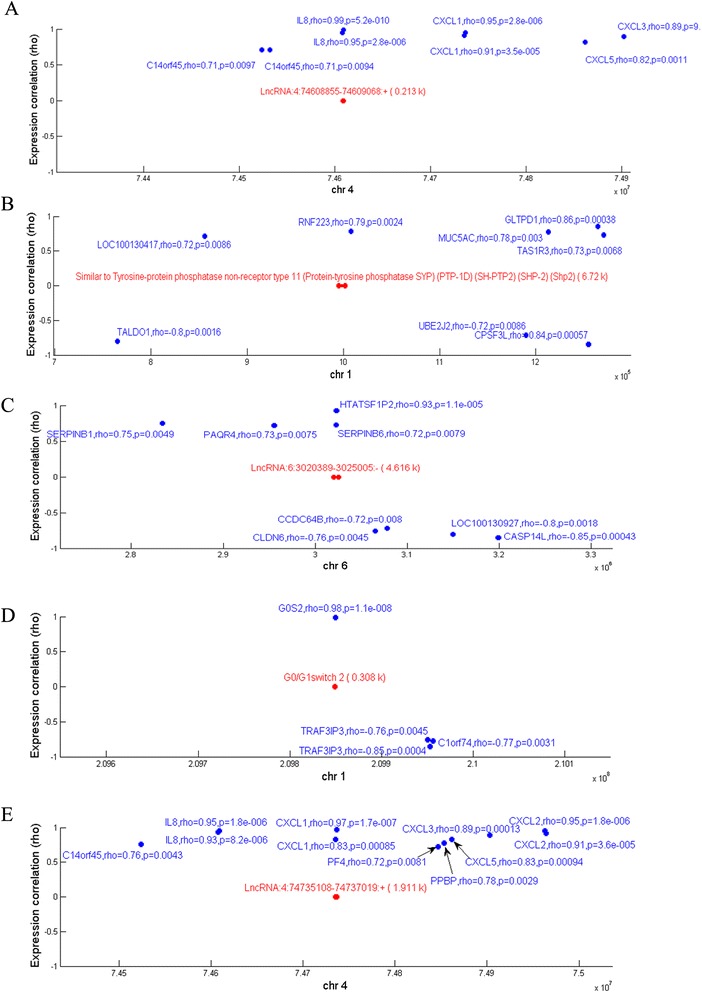
**Representative lncRNAs and the positions of their ‘cis’ genes in the chromosome.** The X abscissa represents the chromosome genome position, and the Y coordinate represents the correlation coefficient of the lncRNAs and the ‘cis’ genes, with a greater correlation coefficient corresponding to a higher position. The red line (or points) indicates the lncRNA genome width, and numbers in parentheses indicate the length. The blue line (or points) indicates the location of the encoding genes; rho values for the encoding genes and lncRNA expression correlation coefficients between the values, as well as the P-values for the correlation coefficients are shown. **A-E**: The results for the lncRNAs oebiotech_11658, oebiotech_12721, oebiotech_21831, oebiotech_11933, and oebiotech_22652, respectively, are shown.

Evidence suggests that several lncRNAs regulate their own transcription in ‘cis’, as well as that of nearby genes, by recruiting remodeling factors to local chromatin [31]. Therefore, we identified the chromosomal co-expression genes 300 kbp upstream and downstream of the differentially expressed lncRNAs to determine potential lncRNA ‘cis’ genes. We searched the genes in the chromosome 300 kbp upstream and downstream of the differentially expressed lncRNAs and identified the common lncRNA co-expression genes within these regions of the chromosome; these genes were considered potential ‘cis’ genes of the lncRNAs. A data reduction strategy was used to overlay the genes located upstream or downstream lncRNAs and co-expression genes. The results of the ‘cis’ analyses were shown in Additional file 4. In MG patients with thymoma versus healthy controls, 157 lncRNAs had 452 ‘cis’ genes. Among these, 4 lncRNAs (oebiotech_11658, oebiotech_12721, oebiotech_21725 and oebiotech_21831) had 8 cis genes, and the lncRNA with the highest aberrant expression, lncRNA oebiotech_11933, had 3 cis genes (C1orf74, G0S2 and TRAF3IP3). The aberrantly expressed lncRNA A_24_P927716 had 2 cis genes (ACSL1 and LOC731424). In MG patients without thymoma versus healthy controls, 36 lncRNAs had 127 ‘cis’ genes upstream or downstream of their chromosomal position: lncRNA oebiotech_22652 had the highest number of ‘cis’ genes and the aberrantly expressed lncRNA oebiotech_11933 had 3 ‘cis’ genes (C1orf74, G0S2, and TRAF3IP3). The lncRNA oebiotech_03926 had 2 ‘cis’ genes (BAALC and AZIN1). In MG patients with versus without thymoma, 58 lncRNAs had 271 ‘cis’ genes upstream or downstream of their chromosomal position: lncRNA oebiotech_22652 had the highest number of ‘cis’ genes, whereas lncRNA oebiotech_12244 had 6 ‘cis’ genes(PPBP, CXCL1, CXCL5, PF4V1, PF4, and C14orf45). The cis relationships of 5 outstanding dysregulated lncRNAs (lncRNA oebiotech_11658, oebiotech_12721, oebiotech_21831, oebiotech_11933, and oebiotech_22652) are shown in Figure 4. According to the ‘cis’ transcriptional regulation mechanism, the genes that regulate transcription in ‘cis’ are affected by other genes whose chromosome positions are not far apart. Thus, the data reduction strategy was used to overlay the genes located upstream or downstream of the lncRNAs and the co-expression genes.

### The ‘trans’ mechanism of the aberrant lncRNAs and the construction of the TF-lncRNA-target gene network

**Figure 5:**
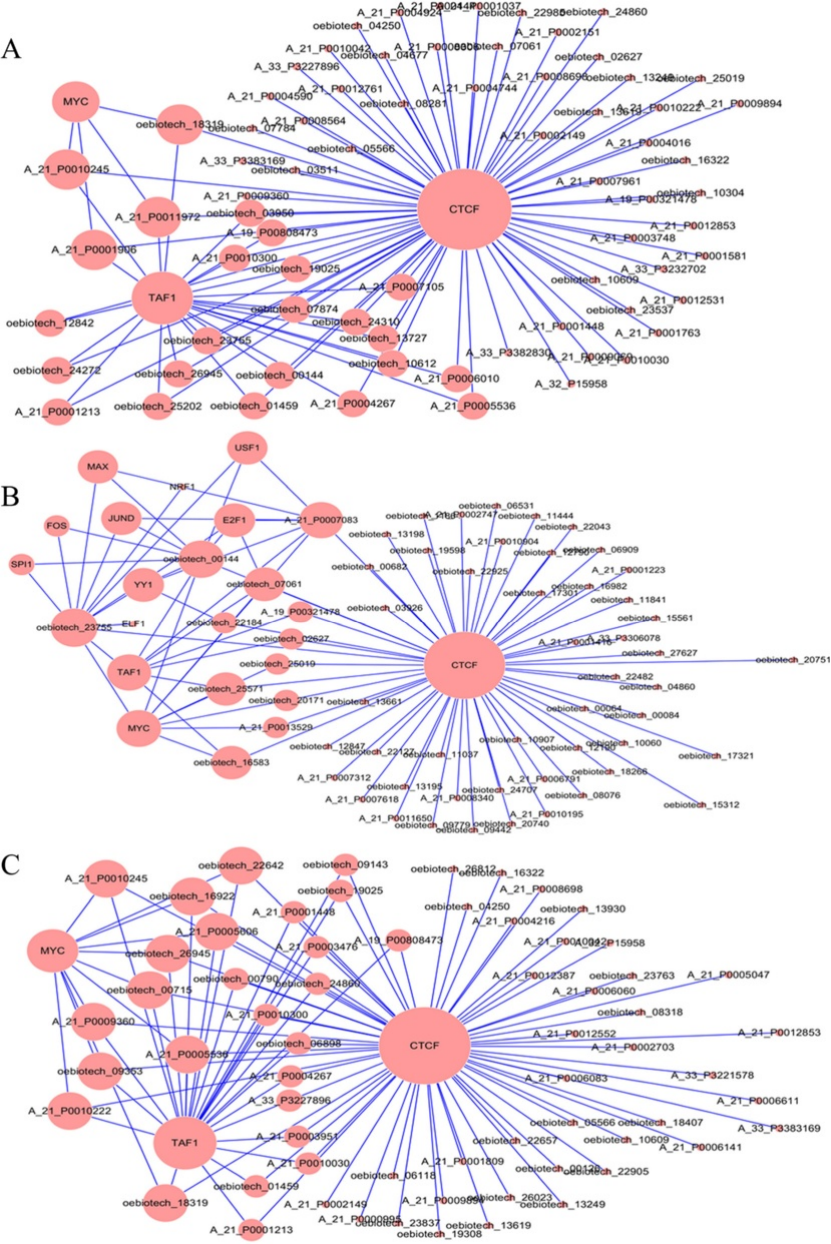
**The “TF-lncRNA” two-element networks for the three groups. A**: The “TF-lncRNA” network between the MG with thymoma and healthy groups. **B**: The “TF-lncRNA” network between the MG without thymoma and healthy groups. **C**: The “TF-lncRNA” network between the MG with thymoma and MG without thymoma groups.

Currently, the known ‘trans’ regulation mechanism involves the factors mediating chromatin regulation and transcription. Using previously described methods [32,33], we calculated the lncRNA co-expression genes of chromatin regulators and transcription factors(TF) in the Encyclopedia of DNA Elements(ENCODE) [34] to identify common genes involved in lncRNA regulation. The “TF-lncRNA” two-element network was generated using Cytoscape software. The “TF-lncRNA” network is large and complex because numerous aberrant lncRNAs are involved. Therefore, we selected the top 100 largest relationships with the “TF-lncRNA” network to generate a core network map (Additional file 5). Figure 5A shows the “TF-lncRNA” core network map for MG patients with thymoma versus healthy controls. The transcription factor CTCF modulated the expression of 72 lncRNAs, whereas the TF TAF1 modulated the expression of 24 lncRNAs, and the TF MYC modulated the expression of 4 lncRNAs. Figure 5B shows the “TF” core network map for MG patients without thymoma versus healthy controls. The transcription factor CTCF modulated the expression of 60 lncRNAs, whereas the TF MYC modulated the expression of 9 lncRNAs. The “TF-lncRNA” core network map for MG patients without thymoma versus MG patients with thymoma indicated that TF TAF1 modulated the expression of 8 lncRNAs (Figure 5C). The transcription factor CTCF modulated the expression of 63 lncRNAs, the TF TAF1 modulated the expression of 26 lncRNAs, and the TF MYC modulated the expression of 11 lncRNAs. These three maps provide a vivid picture of the relationship between the lncRNAs and transcription factors and generate additional information for future studies.

**Figure 6:**
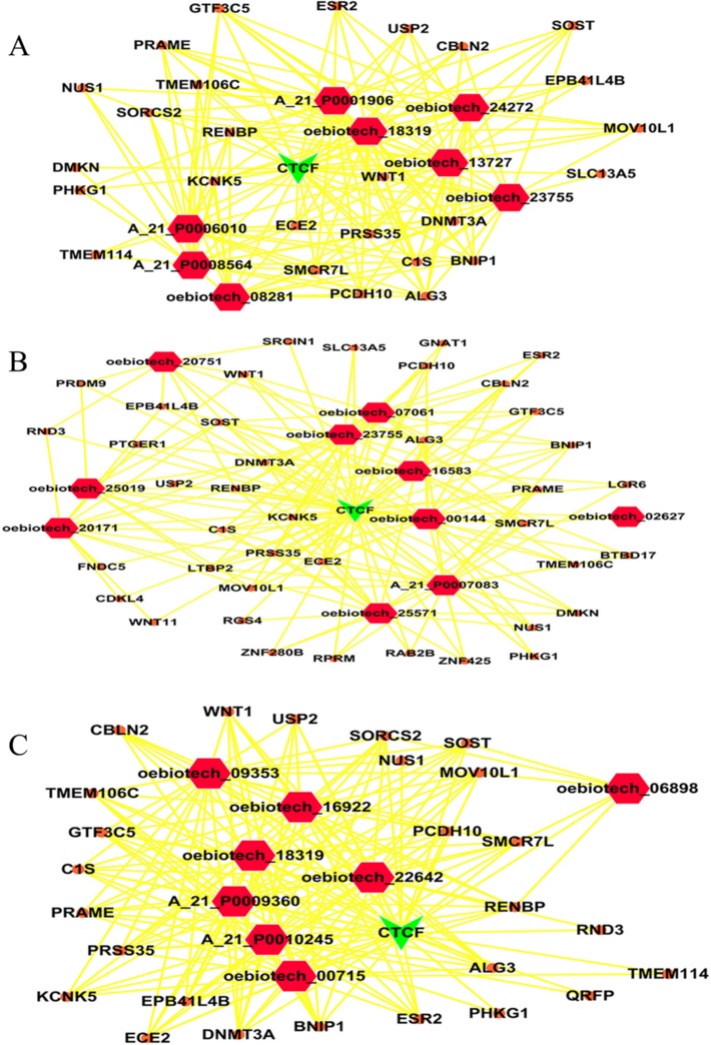
**The “TF–lncRNA-gene” core network map for the three groups. A**: The“TF–lncRNA-gene” core network of the disturbed lncRNA expression in MG patients with thymoma versus healthy controls. **B**: the “TF-lncRNA-gene” core network of the disturbed lncRNA expression in MG patients without thymoma versus healthy controls. **C**: The “TF-lncRNA-gene” core network of the disturbed lncRNA expression in MG patients with thymoma versus MG patients without thymoma.

Based on the results of the lncRNA co-expression analysis, we added the target genes into the “TF-lncRNA” network to determine the “TF-lncRNA-target genes” three-element network relationship. The networks were large and complex; therefore, we selected the top 300 relationships among lncRNAs, TFs and target genes to produce the core network map (Additional file 6). Figure 6A shows the core TF-lncRNA-target gene relationship for MG patients with thymoma versus healthy controls, containing 8 lncRNAs with disrupted expression (lncRNAs oebiotech_24272, oebiotech_23755, oebiotech_18319, oebiotech_13727, oebiotech_08281, A_21_P0008564, A_21_P0006010 and A_21_P0001906), 27 target genes and 1 core TF CTCF in this core map. Figure 6B shows the core TF-lncRNA-target gene relationship in MG patients without thymoma versus healthy controls, containing 10 lncRNAs with disrupted expression (lncRNAsoebiotech_25571, oebiotech_25019, oebiotech_23755, oebiotech_20751, oebiotech_20171, oebiotech_16583, oebiotech_07061, oebiotech_02627, oebiotech_00144 and A_21_P0007083), 41 target genes and 1 core TF CTCF in this core map. Figure 6C shows the core TF-lncRNA-target gene relationship in MG patients with thymoma versus MG patients without thymoma, containing 8 lncRNA with disrupted expression (lncRNAs oebiotech_22642, oebiotech_18319, oebiotech_18319, oebiotech_09353, oebiotech_06898, oebiotech_00715, A_21_P0010245, and A_21_P0009360), 27 target genes and1 core TF CTCF in this core map. The relationship among these three elements could be visualized using these three maps. Similar to the results shown in Figure 6A, the only core TF CTCF association regulated the expression of 8 lncRNAs and 27 target genes. The 8 lncRNAs might also regulate the expression of the 27 target genes. As observed for “CTCF-lncRNA oebiotech_24272-SOST” in this map, target genes, such as SOST, were co-expression genes for lncRNA oebiotech_24272. The transcription factor CTCF may regulate the expression of lncRNA oebiotech_24272and target genes, such as SOST. Thus, these maps provided valuable information concerning transcription factors, lncRNAs and target genes.

## Discussion

The thymus plays a distinct role in the pathogenesis of different MG subtypes. Marx et al. [10] divided MG into subgroups (early onset MG, late onset MG, and thymoma-associated MG) based on age of disease onset, gender and genetic biases, antibody specificities and associated thymus pathologies. However, in the present study, we divided MG patients into two groups: MG with or without thymoma. The first aim was to identify lncRNA subgroups as biomarkers to distinguish MG patients with or without thymoma from healthy individuals. As shown in Table 1 and Figure 2, numerous lncRNA subgroups were identified, providing the first evidence of the involvement of lncRNAs in MG. We selected 12 lncRNAs (lncRNA oebiotech_11933, lncRNA A_24_P927716, lncRNA A_21_P0010030, lncRNA A_21_P0002844, lncRNA oebiotech_11933, lncRNA oebiotech_02627, lncRNA oebiotech_22482, lncRNA A_19_P00315959, lncRNA oebiotech_13222, lncRNA oebiotech_22652, and lncRNA oebiotech_16223) for further validation among the 34 MG patients with or without thymoma. In addition, we identified 42 upregulated and 93 downregulated lncRNAs and 80 upregulated and 43 downregulated mRNAs (Figure 1D and Additional file 2) exhibiting similar expression patterns, suggesting that these lncRNAs and mRNAs are potential biomarkers for the diagnosis of MG. Thus, the identification of a subset of aberrant lncRNAs and mRNAs is the first highlight of the present study.

Precisely annotating the functions of lncRNAs remains complex. Here, we interpret the lncRNA functions based on co-expression gene GO and pathway analyses. As shown in Table 4), lncRNA oebiotech_11933is associated with the MAPK signaling pathway, chemokine signaling pathway, cytokine-cytokine receptor interaction, NOD-like receptor signaling pathway, Toll-like receptor signaling pathway, and adipocytokine-signaling pathway. Colombara et al. [35] reported that the constitutive activation of p38 and ERK1/2 MAPKs in the epithelial cells of a myasthenic thymus leads to IL-6 and RANTES overexpression. Cytokines and chemokines regulate immune responses in inflammatory diseases. Naïve CD4^+^cells differentiate into T helper type(Th1, Th2, Th9, Th17, and Th22) or regulatory T cells (Treg) through the expression of cytokines and chemokines. Because of the responses to chemokines and the interactions of these molecules with other cells, cytokines and chemokines are likely important in the pathogenesis of MG. Cufi et al [30] conducted RT-PCR analyses demonstrating large increases in the expression of interferon (IFN)-I subtypes (IFN-α2, IFN-α8, IFN-ϖ and IFN-β) in thymoma-associated MG, but not in MG patients without thymoma in control thymuses, and the abnormal regulation of dsRNA-sensing molecules, with the increased expression of Toll-like receptor 3 (TLR3) and decreased expression of protein kinase R (PKR) and dsRNA helicases (RIG-I and MDA5) in MG patients with thymoma. These authors suggested that INF-I overexpression and the activation of innate immunity pathways in thymoma-associated MG may develop after a pathogen infection. Cufi et al [27,29] also observed the contribution of IFN-I and IFN-III subtypes in thymic changes associated with MG. The IFN-I and IFN-III subtypes, particularly IFN-β, specifically induce α-AChR expression in thymic epithelial cells (TECs), and IFN-β increases TEC death and the uptake of TEC proteins in dendritic cells. In parallel, these results showed that IFN-β increased the expression of the chemokines CXCL13 and CCL21 in TECs and lymphatic endothelial cells, and similar results were obtained in C57BL/6 mice. All of these studies were performed using thymus tissues. In the present study, our aim was to identify potential biomarkers for the diagnosis of MG; therefore, we performed our studies on PBMCs. We identified several cytokine and chemokine GO terms and pathways that are consistent with the aforementioned studies. It is reported that the cytokines/chemokines in PBMCs may originate from the thymus [7,10]. Uzawa et al. [36] measured the serum levels of 27 cytokines/chemokines in 47 anti-acetylcholine receptor antibody-positive patients with MG and 20 healthy controls to investigate the contribution of cytokines/chemokines in MG pathogenesis. These authors identified eight cytokines that were significantly changed among MG patients with thymoma (i.e., IL-4, IL-8, IL-15, eotaxin, macrophage inflammatory protein-1α, macrophage inflammatory protein-1β, VEGF and IL-1b). Cordiglieri et al. [37] reported that TLR4 stimulation in MG TECs increased CCL17 and CCL22 expression and induced the production of Th17-related cytokines. TLR4 signaling in the MG thymic milieu might affect cell-to-cell interactions, favoring autoreactive T-cell activation. These results and the results obtained in the present study suggest that lncRNA oebiotech_11933 could play an important role in the pathogenesis of MG.

The most enriched GO terms in the predicted target genes of the lncRNAs were ‘cell response to interferon-γ’, ‘platelet degranulation’, ‘chemokine receptor binding’ and ‘cytokine-cytokine interaction’ in the pathogenesis between MG patients with thymoma and MG patients without thymoma (Figure 3). Many studies have shown that the number of immune cells secreting IL-2, IL-4, IFN-γ, and IL-10 are significantly higher in patients with MG than in healthy individuals [38-40]. The results of the present study demonstrated that the most important GO term was ‘cell response to interferon-γ’, suggesting that aberrant lncRNAs may play an important role in regulating IFN-γ expression. Interferon γ is a soluble cytokine and a member of the type II interferon family with antiviral, immunoregulatory and anti-tumor properties as a potent activator of macrophages. Tuzun et al. [41] reported that MG patients with low plasma IL-6 and IFN-γ levels attained better clinical improvement following etanercept treatment. An increasing number of studies have shown that IFN-γ regulates T and B cell differentiation [42-44]. The GO and KEGG pathway analysis of lncRNA co-expression genes to determine the functions of lncRNAs is the second highlight in the present study.

The molecular regulation through lncRNAs remains unknown because the functions of lncRNAs vary [32]. Indeed, lncRNAs have been identified using a variety of methods, and the number of specific lncRNAs shown to influence genomic functions, including roles in imprinting [45], enhancer functions [46,47], X chromosome inactivation [48], chromatin structure [49] and genomic rearrangements during the generation of antibody diversity [50], is increasing. Here, we utilized the ‘cis’ versus ‘trans’ regulatory mechanisms to obtain additional information on dysregulated lncRNAs. In the present study, we explored ‘cis’ regulatory relationships (Additional file 4). The outstanding lncRNAs are shown in Figure 4. The increased expression of cytokines and chemokines, such as IL-8, CXCL1, CXCL3, CXCL3, and CXCL5, was observed among the oebiotech_11658, oebiotech_12721, oebiotech_21831, oebiotech_11933, and oebiotech_22652 ‘cis’ genes, suggesting that these lncRNAs may regulate the expression of these cytokines and chemokines. The results of this ‘cis’ analysis provide additional information concerning the regulation mediated through lncRNAs and the biological processes in the pathogenesis of MG. We constructed the “TF-lncRNA” and “TF-lncRNA-target gene” network based on the results of the ‘trans’ analysis (Additional file 6). The core TF-lncRNA-target gene network (Figures 5 and 6) showed that TFs, including CTCF, TAF1and MYC, regulate lncRNA expression in MG. The CTCF gene is a member of the BORIS + CTCF gene family, encoding a transcriptional regulator protein with 11 highly conserved zinc finger (ZF) domains. This nuclear protein uses different combinations of the ZF domains to bind different DNA target sequences and proteins. Depending on the context of the site, this protein binds to the histone acetyltransferase (HAT)-containing complex and functions as a transcriptional activator or binds to the histone deacetylase (HDAC)-containing complex and functions as a transcriptional repressor. When the protein is bound to a transcriptional insulator element, the communication between enhancers and upstream promoters is blocked, thereby regulating imprinted expression [51,52]. Thus, the ‘trans’ analysis provides another method to interpret lncRNA function and the biological processes in the pathogenesis of MG. The results of the ‘cis’ and ‘trans’ analyses provide information concerning the modular regulation of lncRNAs, representing the third highlight of the present study.

## Conclusion

In the present study, we identified a subset of aberrant lncRNAs to distinguish MG patients with or without thymoma compared with healthy individuals. The function and biological processes of lncRNAs in the pathogenesis of MG were determined according to co-expression gene GO and pathway annotations. The results of the ‘cis’ and ‘trans’ analyses provide information for future studies of aberrantly expressed lncRNAs in MG. These results provide support for future investigations of the pathogenesis of MG.

## Method

### Patients and sample collection

#### MG Patient Demographics

A total of 34 MG patients examined at the Neurology Department of Xiangya Hospital and 13 healthy donors were recruited from May 2010 to March 2012. MG was diagnosed based on a combination of fluctuating muscle weakness with a positive neostigmine test or abnormal single-fiber EMG test. The MG patients were divided into two groups: patients with or without thymoma. The Myasthenia Gravis Foundation of America (MGFA) clinical classification was also used to identify MG subgroups. All MG patients did not receive immunomodulatory or immunosuppressive treatment and were not treated with thymectomy. Information pertaining to sex, age at onset, disease duration, MGFA clinical classification upon first visit to the hospital, additional autoimmune diseases, including thyroid disorders, systemic lupus erythematosus (SLE), rheumatoid arthritis, and Sjogren syndrome, and previous treatments, including acetylcholinesterase inhibitors, immunosuppressive drugs, prednisone, plasma exchange, IVIg and thymectomy, is summarized in Additional file 7. The samples MG1-8 and Normal1-4 were selected for lncRNA chip analysis, and all of the samples in Additional file 7 were used for the validation of lncRNA expression. All patients and healthy volunteers provided informed consent for sample collection and have signed informed consent forms. Collections and use of tissue samples were approved by the Ethical Review Committee of Xiangya Hospital. The PBMCs were isolated from approximately 10 ml of EDTA whole blood using a Ficoll-Paque™ PLUS density gradient, and then, 5x10^7^ cells were resuspended in Trizol® (Invitrogen) and stored at -80°C.This study was approved by the ethical review committees of Xiangya Hospital.

### RNA extract and the lncRNA chip array

#### lncRNA microarrays

Total RNA was extracted from PBMCs using Trizol® reagent (Invitrogen). Approximately 200 ng of total RNA from each sample was used for the lncRNA microarray analysis. lncRNA expression was analyzed from May 2012 to August 2013 using OE_Biotech Human lncRNA chip software V2.0(4*180K),containing 46,506 lncRNAs and 30,656 mRNAs collected from eight authoritative databases, including Agilent_ncRNA, lncRNAdb, Gencode V13, H-invDB, NONCODE v3.0, RefSeq, UCR and UCSC_lncRNAs Transcripts. The lncRNA chip experiments were conducted at the OEbiotech Corporation in Shanghai, P.R. China.

### Bioinformatics analysis

#### Difference lncRNAs and mRNA screen

Raw data from each array were first normalized using GeneSpring software (version 12.5) and subsequently analyzed using an unpaired t-test, with a P-value cut-off of 0.05 and a fold-change cut-off of 2.0.

#### Difference integration analysis (Venn analysis)

MG patients with or without thymoma were compared with each other and healthy controls. The common characteristic elements between the three groups were determined using Venn analysis.

#### Difference lncRNA and mRNA clustering analysis

Different lncRNAs and mRNAs were analyzed using Cluster 3.0 software, and the data were used to examine a series of parameters, such as log transform data, normalized genes and arrays, and hierarchical parameters of genes and arrays. The results were further analyzed using Tree View software. Green-yellow indicates low expression, and red indicates high expression.

#### lncRNA co-expression analysis and gene function annotation

The expression of different lncRNAs and mRNAs was analyzed using Pearson’s correlation coefficient. The absolute value of 0.8 was considered relevant, a value less than 0.8 represented a negative correlation, and a value greater than 0.8 represented a positive correlation. A P-value of less than 0.05 was considered statistically significant. The expression of genes encoding each differentially expressed lncRNA, the ontology classification of the co-expression genes based on gene annotation and summary information are available through DAVID (Database for Annotation, Visualization and Integrated Discovery). The predicted target genes were assigned to functional groups based on molecular function, biological processes and specific pathways. The lncRNA gene function was predicted based on the co-expression gene GO functional annotation, selecting (according to the P-value) the top 100 GO and 200 function reliability prediction terms. Statistical function annotation generated additional GO terms, and the most enriched terms might reflect potential lncRNA functions.

### ‘Cis’ analysis of lncRNAs and co-expression genes

The gene location for different lncRNAs on the chromosome was determined. Subsequently, the common lncRNA co-expression genes were intersected to identify the genes 300 kbp upstream or downstream of the lncRNAs as potential ‘cis’ genes. The schematic shows the chromosome location of the lncRNAs and cis genes.

#### TF-lncRNA network

lncRNA sequences were mapped to the genome in the Sanger database. Jemboss software was used to examine the alignment of lncRNA and putative transcription factor binding sequences. The genome browser database was used to build the network describing the relationships between transcription factors and lncRNAs. An adjacency matrix was implemented in Java according to the binding of lncRNA and transcription factors. The core transcription factor is the most important center in the network, with the highest degree of expression [53,54]. Pearson’s correlation analysis [53] was used to measure the regulatory ability of transcription factors by calculating the correlation between transcription factors and lncRNAs. The TF and lncRNA relationship was generated using Cytoscape software. The red circle indicates the enriched lncRNAs or TFs, and the larger size indicates increasing enrichment.

#### TF-lncRNA-gene network

The TF-lncRNA-gene network was constructed based on the interactions of lncRNAs and target co-expression genes as previously described [55]. The lncRNA co-expression genes and the compounds for chromatin regulation and transcription complexes were intersection elements. Intersection elements might participate in lncRNA-mediated gene regulation. The three groups were generated using Cytoscape software based on the “TF-lncRNA” two-element network. Because many lncRNAs are involved, the “TF-lncRNA” network is difficult and complex. Therefore, we selected the top100 closest associations with the “TF-lncRNA” network to produce the core network map.

### Quantitative reverse transcription–polymerase chain reaction analysis

Total RNA was extracted using Trizol® reagent (Invitrogen). The primers for RT-PCR were designed based on the lncRNA sequences from the UCSC. The primers were synthesized and purified at Invitrogen (Shanghai, China). The RT reactions were performed using a cDNA synthesis kit (Bio-Rad, Hercules, CA). Real-time PCR was performed using the ABI StepOne Plus™ Multicolor. The qPCR cycle was 98°C for 2 min, followed by 40 cycles of 95°C for 15 sec and 60°C for 30 sec. A final melt-curve analysis (60–95°C) was included. The standard curve was produced with slopes at approximately -3.32 (~100% efficiency). The lncRNA PCR results were quantified using the 2^ΔΔct^ method against GAPDH for normalization. The data represent the means of three experiments.

Real-time PCR primers:

- oebiotech_11933-F: GAAACGGTCCAGGAGCTGAT
- oebiotech_11933-R: CTTGCTTCTGGAGAGCCTGT
- A_24_P927716-F: TCTGCCCTTCACCTGCTCCT
- A_24_P927716-R: TTGTAGTAGTTGTCAAAAAT
- A_21_P0010030-F: GCAATGTCAAGGCCTCATCT
- A_21_P0010030-R: CGCTATGGCAAGTCACAAGA
- A_21_P0002844-F: GGAAGGCAATGAATGAGAAG
- A_21_P0002844-R: AGATCAGTAGGAAGTGGTAT
- oebiotech_03926-F: GCGTGGTGGATCACTTCTGT
- oebiotech_03926-R: ACATGGCTTTCATGCTAAAT
- oebiotech_02627-F: GGGATCTCAAACCTGGAACA
- oebiotech_02627-R: TTCGGCATCTCGTTAGCTCT
- oebiotech_22482-F: AACCATAACCAGCCAACCAA
- oebiotech_22482-R: CCTGGGCAACAGAGCAAGAC
- A_19_P00315959-F: GACGGAACCACATGGAGACT
- A_19_P00315959-R: GCAGCTATTGTCTGCCTTCC
- oebiotech_13222-F: TTCAGAATAACCCGCCAGTC
- oebiotech_13222-R: ATTCACCAAGCATGCAAACA
- oebiotech_22652-F: ATGCCAGCCACTGTGATAGA
- oebiotech_22652-R: ACTGACATTCATCTAAACAG
- oebiotech_16223-F: CTCAGCAAAAATGCCCAAAT
- oebiotech_16223-R: GGGAGGTGTAGCTGAAGCAG
- GAPDH-F: GAGTCAACGGATTTGGTCGT
- GAPDH-R: TTGATTTTGGAGGGATCTCG

## Additional files

Additional file 1:
**The difference lncRNAs and mRNAs.**
Additional file 2:
**The same tendency expression lncRNAs and mRNAs.**
Additional file 3:
**The dyexpression lncRNAs annotation.**
Additional file 4:
**The dyexpression lncRNAs cis analysis.**
Additional file 5:
**The lncRNAs and their core TF relationship.**
Additional file 6:
**The lncRNAs-TF-target gen coreNET relationship.**
Additional file 7:
**The samples information.**

## Abbreviations

MG: Myasthenia gravis
lncRNAs: Long non-coding RNAs
GO: Gene Ontology
KEGG: Kyoto encyclopedia of genes and genomes
AChR: Acetylcholine receptor
MuSK: Muscle-specific kinase
LRP4: Lipoprotein-related protein 4
SLE: Systemic lupus erythematosus
ORFs: Open reading frames
TLR3: Toll-like receptor 3
IFN: Interferon
PKR: Protein kinase R
TECs: Thymic epithelial cells
MGFA: Myasthenia Gravis Foundation of America
TFs: Transcription factors
ENCODE: Encyclopedia of DNA Elements
RIG-I: dsRNA helicases
PBMC: Peripheral blood mononuclear cell
